# Source-Prior Engineering for Bayesian Optical Sensing in Time-Reversed Young Interferometry

**DOI:** 10.3390/s26154698

**Published:** 2026-07-23

**Authors:** Jianming Wen

**Affiliations:** Department of Electrical and Computer Engineering, Binghamton University, Binghamton, NY 13902, USA; jwen7@binghamton.edu

**Keywords:** time-reversed Young interferometry, Bayesian optical sensing, source-prior engineering, Fisher information, optical interferometry, fixed-detector sensing, source coding

## Abstract

Time-reversed Young (TRY) interferometry reconstructs interference from a fixed detector by reading out a programmable source-label distribution. This work formulates the architecture as a source-coded Bayesian response sensor. For a perturbation parameter θ, the detected source-label histogram is a posterior distribution determined by a programmed source prior, an optical likelihood for a fixed-detector click, and an evidence factor equal to the click probability. The key point is not the Bayesian identity itself, but its physical implementation: in TRY the prior is imposed before propagation and can therefore reshape the response ensemble actually sampled by the detector. The normalized posterior is shown to respond through a centered likelihood score, and the detected-event Fisher information is the posterior variance of this score. This identifies posterior-weighted score contrast, rather than local response magnitude alone, as the relevant sensing resource. The framework separates posterior-shape information from evidence information, giving a resource-aware way to judge near-null response enhancement. It also yields practical design rules: a two-label source code converts a weak perturbation into a fixed-detector label imbalance, while the multiparameter score covariance provides a route to nuisance rejection and gives a minimal-label rank condition for sensing multiple perturbations. A passive double-slit implementation with weak one-slit phase and loss perturbations is proposed, requiring only fixed-detector source scans before and after calibrated perturbations. Practical tolerances associated with source-programming error, drift, background, imperfect coherence, and polarization mismatch are analyzed, and extensions to multi-aperture and integrated photonic systems are formulated. The results position TRY as a source-programmable Bayesian sensing architecture complementary to conventional detector-plane Young interferometry.

## 1. Introduction

Young’s double-slit experiment is normally a predictive measurement. A known source prepares an optical field, and interference is observed as a detector-plane intensity distribution. In its simplest form, a fixed source coordinate $y_{0}$ produces a normalized response over detector coordinate *x*. By contrast, time-reversed Young (TRY) interferometry changes this statistical arrangement. A detector is fixed at coordinate *d*, while a point-addressable source coordinate *y* is scanned or programmed. The interference pattern is then reconstructed in source-label space rather than by scanning the detector plane [1,2,3].

This reversal is more than a geometric relabeling. In conventional Young interferometry (CYI), the detector coordinate is the output random variable. In TRY, the detected event at *d* conditions the source-label distribution. Thus the measured object is naturally a posterior distribution [4,5,6],

(1) $$P\left(y|d;\theta \right)=\frac{p(y)L(d|y;\theta )}{Z(d;\theta )},$$

where p(y) is the programmed source prior, L(d|y;θ) is the likelihood for a click at the fixed detector, and θ denotes a physical perturbation inside the interferometer. The normalization

(2) Z(d;θ)=∫dyp(y)L(d|y;θ)

is the evidence. Here and throughout, *L* denotes a dimensionless per-source-trial click likelihood satisfying 0≤L≤1. The optical intensity or expected count rate is denoted by λ and is converted to *L* through the calibrated detection model introduced in Section 2. Experimentally, *Z* is therefore the click probability for the chosen source program [5,6].

The appearance of Bayes’ rule is not itself the novelty. Bayesian inference, score functions, Fisher information, and weak-response formulas are standard tools in estimation theory and optical metrology [7,8,9,10,11,12]. The point here is that TRY gives these tools a distinct physical implementation. The prior p(y) is not a post-processing weight applied after detection; it is imposed before propagation through the source dwell time, source brightness, an addressable emitter array, a digital micromirror device, or a spatial light modulator. Changing p(y) changes the ensemble of source labels that actually contributes to fixed-detector clicks. This makes the source plane a programmable statistical resource.

Such a source-programmed fixed-detector viewpoint is conceptually adjacent to coded-aperture imaging, Hadamard-transform optics, single-pixel compressive imaging, and computational ghost imaging, where spatial or statistical information is transferred from detector arrays to programmed illumination or source patterns [13,14,15,16]. But in TRY the programmed variable is the source-label prior entering a fixed-detector posterior rather than an object mask or detector-side postselection [17,18,19,20].

**Figure 1:**
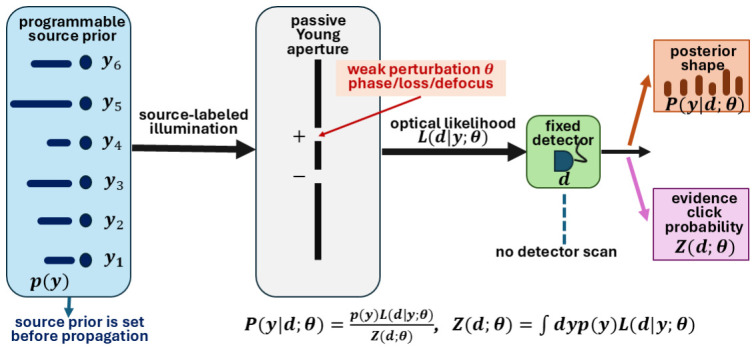
Bayesian response architecture of time-reversed Young interferometry. A programmable source distribution p(y) illuminates a passive Young aperture while the detector is fixed at coordinate *d*. A weak perturbation θ, such as a one-slit phase shift, one-slit loss, or defocus, modifies the optical likelihood L(d|y;θ). The measurement produces two outputs: the normalized posterior shape P(y|d;θ), which carries the source-label response, and the evidence Z(d;θ), which is the click probability for the chosen source program. The essential distinction from conventional Young sensing is that the prior p(y) is imposed before propagation and therefore engineers the response ensemble actually sampled by the fixed detector.

Figure 1 summarizes this architecture. A programmed source prior p(y) prepares the source-label ensemble before propagation through a passive Young aperture. For a fixed detector coordinate *d*, the optical system defines the likelihood L(d|y;θ), while the detected data separate into the posterior shape P(y|d;θ) and the evidence Z(d;θ). This diagram also clarifies the operational difference from CYI: TRY moves the programmable degree of freedom to the source side and leaves the detector fixed [21].

The central question addressed in this work is therefore not whether TRY obeys a Bayesian identity, but whether the Bayesian structure can be used to engineer optical response. Given a weak perturbation such as a one-slit phase shift, one-slit loss, tilt, defocus, or local refractive-index change [2,3,22], the question is how the detected source-label posterior moves and how this motion can be optimized by source programming. The answer is that TRY sensitivity is governed by the posterior variance of the perturbation score [6,7,8]. Consequently, the useful resource is not the largest local response at a single source label, but the posterior-weighted contrast among source labels with different responses.

This distinction has practical consequences. It separates normalized posterior-shape information from evidence, or rate, information [5,6]. It leads to a two-label differential source code that converts a weak perturbation into a fixed-detector source-label imbalance. It also gives a multiparameter covariance rule for choosing source labels that enhance a target perturbation while suppressing nuisance parameters [8,9,23]. These results do not imply that TRY universally outperforms CYI. Rather, the two settings define different statistical experiments: conventional Young samples detector-coordinate scores from a fixed source, whereas TRY samples source-label scores conditioned on a fixed detector and shaped by a programmable prior.

The relationship to earlier TRY studies is clarified as follows. The initial TRY work established fixed-detector source-space interference [21]. Subsequent preliminary studies considered differential source-basis encoding [24], null-constrained operation [25], multi-slit and Talbot-like propagation [26], statistical foundations [27], and tilt–defocus sensing [28] as separate directions. The present journal article consolidates those preliminary elements into a self-contained Bayesian sensor theory and extends them in several ways: it treats the programmed source distribution explicitly as a physical prior; derives the exact separation between posterior-shape information and click/no-click evidence information; connects source-prior design to two-label balancing, multiparameter nuisance rejection, and the minimal-label rank condition; reconstructs a complex path response from complementary phase and loss scores; and provides an end-to-end experimental protocol with quantitative robustness criteria for programming error, drift, background, partial coherence, and polarization mismatch. These combined results and implementation analyses are the distinct contribution of the present article rather than a restatement of any single preliminary study.

The paper is organized as follows. Section 2 defines the CYI and TRY statistical experiments. Section 3 derives the TRY susceptibility and its Fisher information. Section 4 develops the statistical consequences: shape–evidence separation, differential source coding, multiparameter covariance, nuisance rejection, and a minimal-label rank rule. Section 5 gives a passive double-slit realization. Section 6 presents an experimental protocol and a conventional Young control. Section 7 discusses physical meaning, limitations, and complementarity. Detailed algebra that is standard but useful for checking is placed in the Appendix A, Appendix B, Appendix C, Appendix D, Appendix E.

## 2. Optical Model and Statistical Experiments

### 2.1. Passive Scalar Propagation

A one-dimensional scalar paraxial model is considered. The source coordinate is *y*, the aperture coordinate is ξ, and the detector coordinate is *x*. Propagation from the source plane to the aperture plane is described by $U_{1}$, the aperture by *T*, and propagation from the aperture plane to the detector plane by $U_{2}$. A physical perturbation is denoted by θ. It may represent, for example, a one-slit phase shift, one-slit loss, transverse tilt, defocus, or a local refractive-index change [2,3,29,30].

The transition amplitude from source coordinate *y* to detector coordinate *x* is

(3) $$A\left(x,y;\theta \right)=\langle x|U_{2}\left(\theta \right)T\left(\theta \right)U_{1}\left(\theta \right)|y\rangle .$$

Equivalently, in an intermediate-plane representation,

(4) $$A\left(x,y;\theta \right)=\int d\xi \;K_{2}\left(x,\xi ;\theta \right)T\left(\xi ;\theta \right)K_{1}\left(\xi ,y;\theta \right),$$

where $K_{1}$ and $K_{2}$ are the source-to-aperture and aperture-to-detector propagation kernels. Such kernel representations are standard in scalar diffraction theory and in paraxial optical-system propagation [2,3,31]. For free-space paraxial propagation over distance *z*,

(5) $$K_{z}\left(u,v\right)=\frac{\exp(ikz)}{\sqrt{i\lambda z}}\exp\;\left[\frac{ik{\left(u-v\right)}^{2}}{2z}\right],$$

with wavelength λ and wavenumber k=2π/λ. This is the one-dimensional Fresnel propagator used throughout the paraxial approximation [2,3,31].

To distinguish optical intensity or count rate from a probability, define the expected detector count rate

(6) $$\lambda \left(x|y;\theta \right)={\eta \left(x,y\right)|A\left(x,y;\theta \right)|}^{2}+B\left(x\right),$$

where η(x,y) converts optical intensity into detected signal rate and B(x) is the background count rate. For an elementary source trial with a detection gate of duration τ, Poisson photodetection gives the mean count μ(x|y;θ)=τλ(x|y;θ). If the recorded outcome is an on/off click, the dimensionless likelihood used in Equations (1) and (2) is

(7) L(x|y;θ)=1−exp[−μ(x|y;θ)],0≤L<1.

Consequently, *Z* is a valid Bernoulli click probability and 1−Z is the corresponding no-click probability. In the low-occupancy regime μ≪1,

(8) $$L\left(x|y;\theta \right)=\tau \lambda \left(x|y;\theta \right)+O\left(\mu^{2}\right).$$

A common, perturbation-independent factor such as τ cancels from the normalized posterior, susceptibility, and detected-event Fisher information, but it must be retained when evaluating the absolute evidence or throughput. The click-likelihood and count-rate scores are related exactly by

(9) $$\partial_{\theta }\ln L=\frac{\mu }{\exp(\mu )-1}\;\partial_{\theta }\ln\lambda ,$$

so they coincide to leading order for μ≪1. At higher flux, the measured Poisson counts can instead be analyzed directly using the rate model given in Section 4 and Appendix B. This distinction follows the standard intensity-detection model with calibrated efficiency and additive background [29,30,32].

The same optical likelihood underlies CYI and TRY. The distinction between the two settings is statistical: which coordinate is fixed, which coordinate is observed, and which coordinate can be programmed before detection. This distinction is the point at which the present work departs from standard propagation theory and defines a different statistical experiment [21].

### 2.2. Conventional Young Statistical Experiment

In CYI, the source coordinate is fixed at $y_{0}$, and the detector coordinate *x* is the observed random variable. The normalized detector-plane distribution is

(10) $$P_{Y}\left(x|y_{0};\theta \right)=\frac{L(x|y_{0};\theta )}{\int dx\;L(x|y_{0};\theta )}.$$

For a perturbation θ, the detector-coordinate score is

(11) $$s_{Y}\left(x\right)=\partial_{\theta }\ln L\left(x|y_{0};\theta \right).$$

The detector-shape Fisher information is therefore

(12) $$F_{Y}^{\det}={\mathrm{Var}}_{P_{Y}\left(x|y_{0};\theta \right)}\left[s_{Y}\left(x\right)\right].$$

Equation (12) is the typical score-variance form of Fisher information [7,8,9]. It is included to make the comparison precise: CYI can be analyzed by the same score-variance language, but the variance is taken over detector coordinate *x*, while the source coordinate is fixed.

### 2.3. TRY Posterior Statistical Experiment

In TRY, the detector coordinate is fixed at *d*, and the source coordinate is programmed according to p(y), with ∫dyp(y)=1. The detected source-label distribution is the posterior in Equation (1). For a discrete source basis $\left\{y_{j}\right\}$, the prior is a set of programmed weights $p_{j}$, and the posterior becomes

(13) $$P_{j}=\frac{p_{j}L_{j}}{\sum_{k}p_{k}L_{k}},\;L_{j}=L\left(d|y_{j};\theta \right).$$

Experimentally, $P_{j}$ is estimated by the normalized fixed-detector count histogram over source labels. This is the discrete experimental form of the Bayesian update, with source-label weights playing the role of a physically programmed prior [4,5,6,21].

This is where TRY differs operationally from CYI. In CYI, *x* is an output coordinate generated by the optical field. In TRY, *y* is an input label selected before propagation. Therefore p(y) is a physical control variable. Changing p(y) changes which source labels contribute to the detected ensemble, rather than merely reweighting data after measurement. This pre-detection control is the statistical resource used in the susceptibility and source-code design developed below.

## 3. Bayesian Susceptibility and Fisher Information

The response of the TRY posterior to a weak physical perturbation is considered next.

Let

(14) $$s_{\theta }\left(y|d\right)=\partial_{\theta }\ln L\left(d|y;\theta \right)$$

be the likelihood score for source label *y* at fixed detector coordinate *d*. The score measures the fractional response of the optical likelihood to the perturbation, as in standard likelihood-based estimation theory [7,8,23].

The TRY susceptibility is defined as the logarithmic response of the normalized source-label posterior,

(15) $$\chi_{T}\left(y|d;\theta \right)=\partial_{\theta }\ln P\left(y|d;\theta \right).$$

Using Equation (1), and assuming that the programmed prior is independent of the perturbation, one obtains

(16) $$\chi_{T}\left(y|d;\theta \right)=s_{\theta }\left(y|d\right)-{\langle s_{\theta }\rangle }_{P},$$

where ${\langle \cdot \rangle }_{P}$ denotes averaging over the detected posterior. The derivation is given in Appendix A.

Equation (16) is a standard centered-score identity for a normalized parametric distribution, but its physical meaning in TRY is important. The normalized posterior does not respond to the absolute likelihood score at one source label. It responds to the difference between that local score and the evidence-averaged score. A perturbation that multiplies all likelihoods by the same fractional factor changes the total click rate but leaves the normalized posterior shape unchanged.

The detected-event Fisher information carried by the posterior shape is

(17) $$F_{T}^{\det}={\mathrm{Var}}_{P}\left[s_{\theta }\left(y|d\right)\right].$$

Thus the sensitivity of the normalized TRY source-label distribution is governed by the posterior variance of the perturbation score, the usual Fisher-information form for a normalized measurement distribution [7,8,9]. Useful posterior-shape sensing requires score heterogeneity across the detected source ensemble. A single source label, even one with a large local score, carries no shape information by itself. Conversely, two or more source labels with different scores can yield a strong posterior response, provided that they retain sufficient posterior weight.

This distinction is especially important near destructive interference. In many two-path settings, a local score can become large near a null because the total amplitude is small. Similar near-null response enhancement is familiar from weak-value and postselected metrology, where large conditional responses must be weighed against postselection probability and technical noise [10,11,12,33,34]. However, the same null can suppress the likelihood and hence reduce the evidence. The useful operating point is therefore not necessarily the darkest fringe. It is the region where the score contrast and detected posterior weight jointly maximize Equation (17), subject to the throughput constraints discussed below. Related optical-sensor implementations of weak-response amplification have appeared in weak-measurement-based biosensing and chiral sensing, although the present TRY mechanism uses source-prior engineering rather than detector-side postselection [35,36].

**Figure 2:**
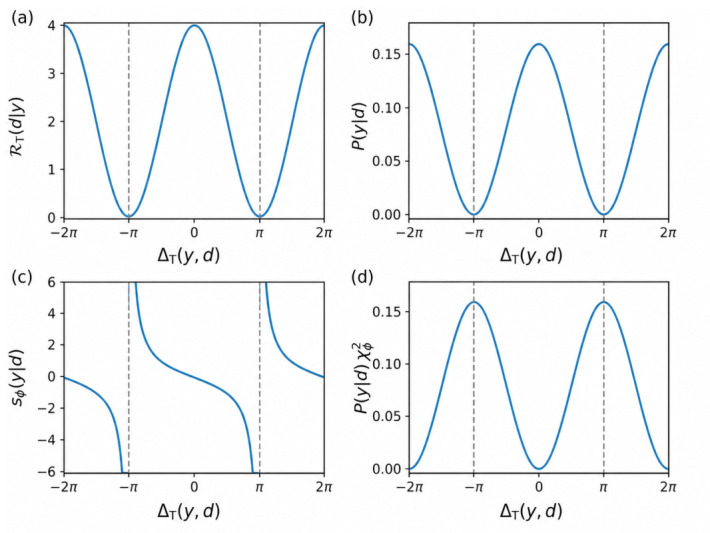
Source-space response landscape for the balanced double-slit TRY model in the low-occupancy regime. (**a**) Relative fixed-detector count-rate profile $R_{T}\left(d|y\right)=4\cos^{2}\left[\Delta_{T}\left(y,d\right)/2\right]$, for which the click likelihood is $L≃\kappa R_{T}$ with a calibrated κ≪1. (**b**) Detected posterior $P\left(y|d\right)=R_{T}\left(d|y\right)/\left(8\pi \right)$ for a uniform source prior over the displayed phase interval; the common factor κ cancels. (**c**) Phase score $s_{\phi }\left(y|d\right)=-\tan\left[\Delta_{T}\left(y,d\right)/2\right]$, clipped for display near the source-space nulls. (**d**) Information density $P\left(y|d\right){\left[s_{\phi }\left(y|d\right)-{\langle s_{\phi }\rangle }_{P}\right]}^{2}=\sin^{2}\left[\Delta_{T}\left(y,d\right)/2\right]/\left(2\pi \right)$. The figure shows that near-null response enhancement must be judged together with posterior weight: the local score can grow strongly near a null, while the experimentally useful information density remains finite.

Figure 2 makes this tradeoff explicit for the balanced double-slit TRY model. Panel (a) shows the relative fixed-detector count-rate profile $R_{T}\left(d|y\right)=4\cos^{2}\left[\Delta_{T}\left(y,d\right)/2\right]$. In the low-occupancy regime, the physical click likelihood is $L≃\kappa R_{T}$, where $\kappa =\tau \lambda_{0}$ contains the calibrated rate scale and gate duration. The profile gives the source-space fringe sampled by the programmed source labels. $\Delta_{T}\left(y,d\right)$ is the relative phase between the lower and upper optical pathways and is defined in Equation (33). Panel (b) shows the corresponding posterior P(y|d) for a uniform source prior over the displayed phase interval; in this ideal case it has the same fringe shape as the rate profile because the common factor κ cancels, but it is normalized as a detected-event distribution. Panel (c) shows the one-slit phase score $s_{\phi }\left(y|d\right)=-\tan\left[\Delta_{T}\left(y,d\right)/2\right]$, whose magnitude increases near the source-space nulls because a small phase perturbation strongly changes the relative two-path cancellation. Panel (d) plots the information density $P\left(y|d\right){\left[s_{\phi }\left(y|d\right)-{\langle s_{\phi }\rangle }_{P}\right]}^{2}$, which remains finite because the posterior weight decreases where the local score grows. Thus the useful sensing region is not identified by the largest local score alone; it is identified by the overlap between appreciable detected probability and large centered-score contrast. This is the same statistical caution underlying Fisher-information analyses of amplified or postselected measurements, but here the conditional ensemble is engineered through the source prior rather than through detector-side postselection [21,33,34].

## 4. Statistical Consequences of the TRY Posterior

The posterior form of TRY has consequences beyond the statement that the source prior is programmable. It changes how perturbation information is organized. In CYI, one usually analyzes a detector-plane distribution generated by a fixed source. In TRY, the detected event defines a posterior ensemble of source labels. This posterior ensemble has a shape, an evidence, and a programmable support. These three features lead to useful statistical design rules [5,6,8,23].

### 4.1. Posterior-Shape Information and Evidence Information

The susceptibility in Equation (16) describes the response of the normalized posterior shape. Because it is a centered score, it is insensitive to a perturbation that multiplies all source-label likelihoods by the same fractional factor. Such a perturbation does not reshape the detected source-label histogram. It only changes the total probability of obtaining a fixed-detector click. This is the usual distinction between normalized distributional information and rate, or yield, information in likelihood-based measurements [8,23,33,34].

The latter effect is carried by the evidence. Its logarithmic response is

(18) $$\partial_{\theta }\ln Z={\langle s_{\theta }\rangle }_{P}.$$

Thus, TRY naturally separates perturbation information into posterior-shape information and evidence, or rate, information. If total intensity is normalized away to reject source-power drift, detector-gain drift, or slow coupling fluctuations, then only the posterior-shape channel is retained. If the absolute click rate is stable and calibrated, the evidence can be used as an additional signal. This separation is also standard in experimental design, where one distinguishes information in the conditional distribution from information in event probability or acquisition rate [5,6].

For a discrete source program, a single source trial either produces a fixed-detector click with recorded label *j*, or produces no click. The per-trial Fisher information is

(19) $$F_{\mathrm{trial}}=ZF_{T}^{\det}+\frac{Z{\langle s_{\theta }\rangle }_{P}^{2}}{1-Z}.$$

Its derivation is given in Appendix B. Equation (19) applies specifically to the gated Bernoulli click model of Equation (7). The first term is the click-probability-weighted information contained in the normalized posterior shape, and the second term is the information carried by the total click/no-click probability. If the experiment instead records unthresholded Poisson counts with source-exposure fractions $p_{j}$, the Fisher information per unit exposure is

(20) $$F_{\mathrm{Pois}}^{\left(\mathrm{rate}\right)}=\sum_{j}p_{j}\frac{{\left(\partial_{\theta }\lambda_{j}\right)}^{2}}{\lambda_{j}}=\bar{\lambda }\left[F_{T,\lambda }^{\det}+{\langle r_{\theta }\rangle }_{P_{\lambda }}^{2}\right],$$

where $\bar{\lambda }=\sum_{j}p_{j}\lambda_{j}$, $P_{\lambda ,j}=p_{j}\lambda_{j}/\bar{\lambda }$, $r_{\theta }=\partial_{\theta }\ln\lambda_{j}$, and $F_{T,\lambda }^{\det}={\mathrm{Var}}_{P_{\lambda }}\left(r_{\theta }\right)$. Thus the same posterior-shape variance appears in both models; only the rate-information term differs. In the low-occupancy limit, Equation (19) reduces to $F_{\mathrm{trial}}=\tau F_{\mathrm{Pois}}^{\left(\mathrm{rate}\right)}+O\left[{\left(\tau \bar{\lambda }\right)}^{2}\right]$. The same decomposition underlies Fisher-information analyses of postselected or lossy measurements, where conditional sensitivity must be assessed together with success probability [33,34,37].

When the rate channel is deliberately discarded, or when it is too noisy to be trusted, the relevant resource-aware shape metric is

(21) $$\Phi_{T}=ZF_{T}^{\det}.$$

This quantity prevents a common overinterpretation of near-null response enhancement. Near destructive interference, a local score may become very large, but the likelihood and hence the click probability may become very small. A large conditional response is experimentally valuable only if enough detected events remain to estimate it. This is the practical reason for reporting both conditional Fisher information and throughput-weighted information. For direct Poisson-count operation, the analogous posterior-shape information rate is $\bar{\lambda }F_{T,\lambda }^{\det}$, rather than $ZF_{T}^{\det}$.

### 4.2. Differential Source Coding

The simplest nontrivial TRY source code uses two source labels, $y_{a}$ and $y_{b}$. Let their detected posterior weights be $P_{a}$ and $P_{b}$, and let their perturbation scores be $s_{a}$ and $s_{b}$. The detected-event Fisher information then reduces to

(22) $$F_{T}^{\det}=P_{a}P_{b}{\left(s_{a}-s_{b}\right)}^{2}.$$

This expression makes the role of score heterogeneity explicit. A single high-response label is not enough. The posterior must contain at least two labels whose scores are different. For fixed scores, Equation (22) is maximized by balanced detected posterior weights, giving

(23) $$F_{T,\max}^{\det}=\frac{{\left(s_{a}-s_{b}\right)}^{2}}{4}.$$

This is a standard variance result, but in TRY it becomes an optical design rule: choose source labels with large perturbation-score separation, then program the source prior so that both labels contribute comparably to the detected posterior. This is closely related in spirit to optimal experimental design, where measurement settings are chosen to maximize an information criterion rather than sampled passively [6,38,39,40].

If the two labels have likelihoods $L_{a}$ and $L_{b}$, balanced detected posterior weights are obtained by

(24) $$\frac{p_{a}}{p_{b}}=\frac{L_{b}}{L_{a}}.$$

This equation uses the calibrated per-trial click probabilities. In the low-occupancy regime, $L_{j}≃\tau \lambda_{j}$, so the same ratio can be obtained directly from background-corrected count rates. If click occupancy is not small, raw intensities or rates should first be converted using Equation (7). For an unthresholded Poisson-count implementation, balancing detected counts instead requires $p_{a}/p_{b}=\lambda_{b}/\lambda_{a}$. For a two-label-only program, the resulting evidence is

(25) $$Z_{ab}=\frac{2L_{a}L_{b}}{L_{a}+L_{b}}.$$

Equation (25) displays the throughput cost of balancing the posterior. It is the TRY version of a familiar design tradeoff: information can be concentrated in selected outcomes, but the acquisition probability of those outcomes remains part of the experimental resource budget [5,33,34]. That is, if one label lies near a null, its score may be large, but its likelihood is small. It must then be assigned a larger prior weight, or longer dwell time, to appear with comparable frequency among detected events.

**Figure 3:**
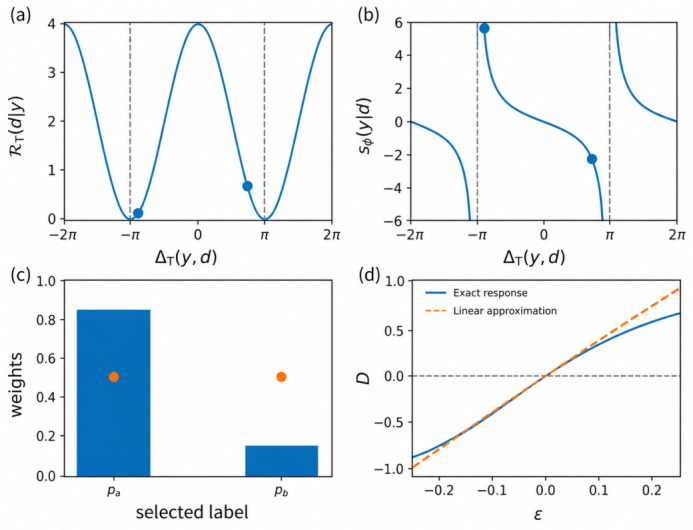
Two-label source-prior engineering in TRY. (**a**) A preliminary fixed-detector source scan gives the relative count-rate landscape $R_{T}\left(d|y\right)$, from which two source labels $y_{a}$ and $y_{b}$ are selected. (**b**) The same labels are chosen to have strong phase-score contrast $s_{a}-s_{b}$, where $s_{j}=s_{\phi }\left(y_{j}|d\right)$. (**c**) Because the two labels generally have different detected rates and hence different click likelihoods, the programmed prior weights $p_{a},p_{b}$ are chosen according to $p_{a}/p_{b}=L_{b}/L_{a}$, producing balanced detected posterior weights $P_{a}=P_{b}=1/2$. (**d**) A weak one-slit phase perturbation ϵ is then read out as the differential label imbalance $D=\left(n_{a}-n_{b}\right)/\left(n_{a}+n_{b}\right)$. The dashed line shows the linear response $D≃[\left(s_{a}-s_{b}\right)/2]\epsilon$.

Figure 3 illustrates the full two-label design logic. Panel (a) shows the relative fixed-detector count-rate landscape $R_{T}\left(d|y\right)$, from which two source labels $y_{a}$ and $y_{b}$ are selected. The labels need not have equal likelihoods; in fact, one may deliberately choose a lower-likelihood label if it provides a stronger perturbation response. Panel (b) shows the corresponding phase-score landscape $s_{\phi }\left(y|d\right)$, where the same two labels are chosen to produce a large score difference $s_{a}-s_{b}$. Panel (c) then shows the essential source-prior correction: unequal programmed weights $p_{a}$ and $p_{b}$ compensate unequal likelihoods so that the detected posterior weights become balanced, $P_{a}=P_{b}=1/2$. Panel (d) shows the resulting differential readout, where a weak one-slit phase perturbation is converted into a label imbalance *D*. Thus Figure 3 turns Equations (24) and (25) into an operational protocol: select labels by score contrast, balance them by dwell time, and estimate the perturbation from a fixed-detector count ratio. Unlike detector-side postselection, this balancing is imposed before propagation by changing the source dwell times.

With detected counts $n_{a}$ and $n_{b}$, define the label imbalance

(26) $$D=\frac{n_{a}-n_{b}}{n_{a}+n_{b}}.$$

To first order in the perturbation,

(27) $$\partial_{\theta }D=2P_{a}P_{b}\left(s_{a}-s_{b}\right).$$

At balanced detected posterior weights, $\partial_{\theta }D=\left(s_{a}-s_{b}\right)/2$. Thus the perturbation is converted into a fixed-detector source-label imbalance. Because *D* is a ratio, common detector gain fluctuations, acquisition-time fluctuations, and source-power drifts that affect both labels similarly are partially canceled. Such differential normalization is a standard strategy for suppressing common-mode technical noise in optical sensing and precision measurement [41,42].

The two-label code is therefore more than a minimal Fisher-information example. It is a practical fixed-detector differential sensor. A preliminary scan identifies candidate labels with large score separation and acceptable likelihood. A second measurement uses only those labels, with dwell times chosen from Equation (24). The perturbation is then estimated from the imbalance *D*, rather than from a full source scan.

### 4.3. Multiparameter Score Covariance and Nuisance Rejection

The same posterior-score structure extends directly to multiple perturbations. Let $\theta =(\theta_{1},\theta_{2},\ldots ,\theta_{m})$ and define

(28) $$s_{\mu }\left(y\right)=\partial_{\theta_{\mu }}\ln L\left(d|y;\theta \right).$$

The posterior-shape Fisher matrix is the score covariance,

(29) $$F_{\mu \nu }^{T}={\mathrm{Cov}}_{P}\left(s_{\mu },s_{\nu }\right).$$

This is the standard Fisher-information matrix for a multiparameter likelihood model [7,8,9,23]. Each source label carries a response vector $s\left(y\right)=(s_{1}\left(y\right),s_{2}\left(y\right),\ldots ,s_{m}\left(y\right))$. A source program selects which response vectors are represented in the detected posterior. Source-prior engineering is therefore equivalent to shaping the cloud of detected score vectors. This covariance view also connects the TRY Fisher matrix to the information-geometric interpretation of statistical models, where local distinguishability is encoded by the Fisher metric [43]. This gives a geometrical interpretation of source-code design analogous to information-matrix design in multiparameter estimation [9,39].

**Figure 4:**
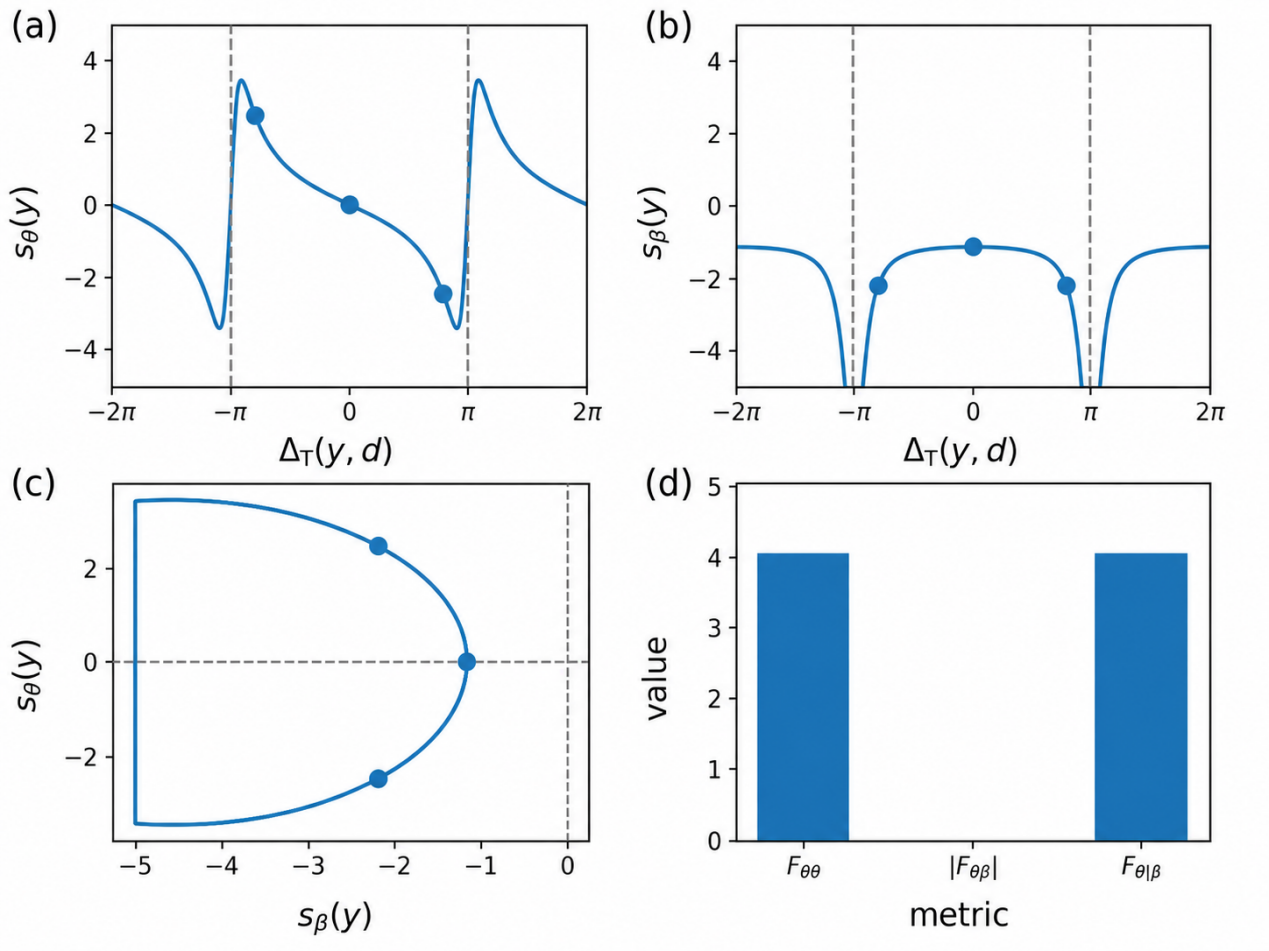
Multiparameter source-prior design in TRY. (**a**) Target phase score $s_{\theta }\left(y\right)=s_{\phi }\left(y|d\right)$ for a slightly imbalanced two-path model. (**b**) Nuisance loss score $s_{\beta }\left(y\right)=s_{l}\left(y|d\right)$ over the same source-label coordinate. (**c**) Score-vector trajectory $\left(s_{\beta }\left(y\right),s_{\theta }\left(y\right)\right)$, showing how selected labels populate the two-dimensional score space. (**d**) Covariance metrics for the selected three-label code: target information $F_{\theta \theta }^{T}$, cross covariance $|F_{\theta \beta }^{T}|$, and nuisance-eliminated information $F_{\theta |\beta }$. The red points mark the selected source labels. The figure shows how a source code can retain target sensitivity while suppressing target–nuisance covariance.

Figure 4 visualizes this covariance design principle for a two-parameter example. Panel (a) shows the target phase score $s_{\theta }\left(y\right)=s_{\phi }\left(y|d\right)$ across source-label phase coordinate $\Delta_{T}\left(y,d\right)$, while panel (b) shows the nuisance loss score $s_{\beta }\left(y\right)=s_{l}\left(y|d\right)$ for the same labels. The red points mark three selected source labels, which sample the two score landscapes differently. Panel (c) replots the same selected labels as score vectors $\left(s_{\beta },s_{\theta }\right)$; this representation makes the Fisher matrix geometrical, because $F_{\mu \nu }^{T}$ is the posterior covariance of this score cloud. Panel (d) shows the resulting target information, cross covariance, and nuisance-eliminated information. The purpose is not merely to maximize the spread of $s_{\theta }$, but to choose labels whose score vectors keep the target direction distinguishable from the nuisance direction. This distinction is important because large single-parameter information can still lead to poor usable sensitivity if it is strongly correlated with nuisance responses.

This is especially useful when one parameter is the desired signal and another is a nuisance. For a target parameter θ and a nuisance parameter β, the information remaining after eliminating the nuisance is

(30) $$F_{\theta |\beta }=F_{\theta \theta }-\frac{F_{\theta \beta }^{2}}{F_{\beta \beta }}.$$

A good source code should produce a large target-score variance while reducing covariance with nuisance scores. The same Schur-complement form appears in multiparameter estimation when nuisance parameters are eliminated from the Fisher matrix [8,9,23]. In practical terms, this means choosing source labels for which the desired perturbation changes the posterior shape in a direction distinguishable from loss, source-power drift, tilt, defocus, or other unwanted changes.

Table 1 classifies common disturbances by the way they enter the likelihood and by the score that they generate. The table is deliberately written at the likelihood level, so the same classification applies to a free-space aperture, a structured mask, or a calibrated integrated photonic transfer function. A common multiplicative fluctuation has a label-independent score and therefore appears only in the evidence channel. By contrast, path-dependent loss, phase drift, visibility loss, background, defocus, and pointing errors produce label-dependent scores and must either be estimated jointly or suppressed by source-code design.

If the posterior occupies *J* source labels, the centered score covariance a has rank of J−1 at most:

(31) $$\mathrm{rank}(F^{T})\leq J-1.$$

Therefore, posterior-shape sensing of *m* independent parameters requires at least m+1 occupied source labels. One label gives no shape information. Two labels can sense only one independent perturbation direction. Three labels are the minimum for two independent directions. This rank statement is a discrete measurement-design constraint rather than a property of the optical aperture itself. This rule is simple but practically important: few-label TRY sensing is possible, but the number of occupied labels limits the number of independent perturbations that can be distinguished from posterior shape alone.

### 4.4. Statistical Complementarity with CYI

The statistical distinction between TRY and CYI is now sharper. CYI also has a score-variance description, but it samples a detector-coordinate score landscape generated by a fixed source. TRY samples a source-label score landscape conditioned on a fixed detector event and shaped by a programmable prior. This is consistent with the source-space reconstruction viewpoint of TRY [21], but here it is expressed as a comparison of statistical experiments rather than as a propagation symmetry.

These two landscapes need not carry the same perturbation information. A perturbation may produce a nearly constant detector-coordinate score in a CYI measurement, while producing strong score contrast across source labels in TRY. The reverse can also occur. The comparison is therefore not a universal sensitivity ranking. It is a comparison between two statistical projections of the same passive optical kernel. This avoids the common pitfall of comparing only local amplification factors without accounting for sampling probability, noise, and measurement architecture [33,34,37].

This complementarity is useful. CYI is naturally suited to detector-plane predictive sensing. TRY is naturally suited to source-posterior response sensing. Its distinctive advantage is that the source side can be programmed before detection. This enables posterior-shape design, differential source coding, and source-side nuisance rejection with a fixed detector. In applications where detector scanning, detector arrays, or detector flat-field calibration are costly or unstable, this source-side architecture can provide a practical alternative. The relevant performance comparison is therefore architecture-dependent and should include throughput, calibration stability, and available source-side programmability. This emphasis on a physically meaningful sensitivity metric is consistent with recent optical-sensing work that formulates sensor performance through information-theoretic or physics-based figures of merit [44].

### 4.5. Comparison with Alternative High-Sensitivity Architectures

Table 2 places TRY beside representative modern optical-measurement strategies. Phase-shifting and heterodyne interferometers provide accurate phase recovery but require phase stepping, a local oscillator, or quadrature calibration [45,46,47,48]. Dark-port and weak-value methods can enlarge a conditional response and can be advantageous under detector saturation, but their performance depends on postselection probability and technical noise [33,34,37]. Single-pixel and computational imaging transfer spatial resolution from a detector array to programmed masks and numerical reconstruction [13,19,49]. TRY is closest to the latter in hardware allocation, but its measured object is a detector-conditioned source-label posterior and its design variable is the physical source prior. Accordingly, the proposed method should be evaluated as an alternative measurement architecture, not as a universal sensitivity enhancement.

## 5. Double-Slit Realization

The general Bayesian response theory is now specialized to a passive double-slit system. This case is analytically transparent and gives a direct experimental test of the proposed source-prior engineering protocol. This case also connects the present framework to the standard two-path interference model while allowing the response quantities to be written in closed form [1,2,3,32].

### 5.1. Two-Path TRY Likelihood

Consider two narrow slits at $\xi_{\pm }=\pm a/2$, with equal transmission amplitudes. The source-to-slit and slit-to-detector distances are $z_{1}$ and $z_{2}$, respectively. Ignoring common prefactors that cancel in normalized probabilities, the two path amplitudes are

(32) $$A_{\pm }\left(x,y\right)=\exp\;\left\{ik\left[\frac{{\left(\xi_{\pm }-y\right)}^{2}}{2z_{1}}+\frac{{\left(x-\xi_{\pm }\right)}^{2}}{2z_{2}}\right]\right\}.$$

The use of two narrow-path amplitudes is the usual point-aperture limit of scalar diffraction theory [2,3,29]. At a fixed detector coordinate *d*, the TRY amplitude is $A\left(d,y\right)=A_{+}\left(d,y\right)+A_{-}\left(d,y\right)$. The relative phase between the lower and upper paths is

(33) $$\Delta_{T}\left(y,d\right)=\arg\frac{A_{-}\left(d,y\right)}{A_{+}\left(d,y\right)}=ka\left(\frac{y}{z_{1}}+\frac{d}{z_{2}}\right).$$

The resulting relative phase has the same linear source–detector structure as the ordinary double-slit phase, but here it is read by varying the source coordinate at fixed detector position. The dimensionless relative count-rate profile is

(34) $$R_{T}\left(d|y\right)={\left|A_{+}\left(d,y\right)+A_{-}\left(d,y\right)\right|}^{2}=4\cos^{2}\;\left[\frac{\Delta_{T}\left(y,d\right)}{2}\right].$$

Writing the detected rate as $\lambda \left(d|y\right)=\lambda_{0}R_{T}\left(d|y\right)+B$, the actual per-trial click likelihood is obtained from Equation (7). In the ideal low-occupancy, background-free limit, $L\left(d|y\right)≃\kappa R_{T}\left(d|y\right)$, with $\kappa =\tau \lambda_{0}\ll 1$. Equation (34) is the source-space TRY fringe. This is the basic source-space reconstruction feature of TRY [21]. The detector is not scanned; the interference is observed by changing the source label *y*. The fixed detector coordinate *d* shifts the phase origin, while the source coordinate scans the phase.

This model is intentionally ideal. In an experiment, unequal slit transmission, finite slit width, background, and imperfect coherence modify the likelihood envelope and reduce the fringe contrast. But these effects do not change the Bayesian structure. They simply replace Equation (34) by the calibrated count-rate profile and the associated click likelihood measured in the baseline source scan. Such corrections are standard in real interferometers and are naturally absorbed into the calibrated likelihood used by the Bayesian response analysis [30,32].

### 5.2. Weak One-Slit Phase Perturbation

Apply a weak phase ϵ to the upper slit,

(35) $$A_{+}\left(d,y\right)\to e^{-i\epsilon }A_{+}\left(d,y\right),\;\left|\epsilon \right|\ll 1.$$

One-path phase perturbations are the standard local probe in interferometric phase sensing [41,42]. Define the upper-path complex weight

(36) $$w_{+}\left(y|d\right)=\frac{A_{+}\left(d,y\right)}{A_{+}\left(d,y\right)+A_{-}\left(d,y\right)}.$$

Quantities of this form are closely related to local weak values and conditional path amplitudes, where a path projector is normalized by the total postselected amplitude [10,11,12,50,51]. The closed-form two-path scores below are count-rate scores and, by Equation (9), are also the click-likelihood scores to leading order in the low-occupancy regime. Thus,

(37) sϕ(y|d)=∂ϵlnλϵ(d|y)ϵ=0≃∂ϵlnLϵ(d|y)ϵ=0=2Imw+(y|d)

as derived in Appendix D. Here the phase score is the imaginary part of the conditional path weight, consistent with the usual role of imaginary weak values as shifts in the conjugate response variable [11,12]. For the balanced two-slit model,

(38) $$w_{+}\left(y|d\right)=\frac{1}{1+e^{i\Delta_{T}\left(y,d\right)}}=\frac{1}{2}-\frac{i}{2}\tan\;\left[\frac{\Delta_{T}\left(y,d\right)}{2}\right],$$

and therefore

(39) $$s_{\phi }\left(y|d\right)=-\tan\;\left[\frac{\Delta_{T}\left(y,d\right)}{2}\right].$$

Near a source-space dark point, $\Delta_{T}=\pi -\delta$ with |δ|≪1,

(40) $$s_{\phi }\left(y|d\right)≃-\frac{2}{\delta },\;R_{T}\left(d|y\right)≃\delta^{2},\;L\left(d|y\right)≃\kappa \delta^{2}.$$

This sensitivity–probability tradeoff is the same caution that appears in weak-value amplification and near-dark-port interferometry [33,34,37,52]. Equation (40) implies that the local score is enhanced as the null is approached, but the likelihood decreases quadratically. Hence the best sensing region is not the exact null. It is a region near the null where the score is large while the detected posterior weight remains sufficient.

### 5.3. Weak One-Slit Loss Perturbation

Apply instead a weak amplitude loss α to the upper slit,

(41) $$A_{+}\left(d,y\right)\to e^{-\alpha }A_{+}\left(d,y\right),\;0<\alpha \ll 1.$$

Amplitude loss provides the complementary perturbation to phase because it probes the in-phase, or real, component of the conditional path response. The corresponding low-occupancy likelihood score, equivalently the count-rate score, is

(42) sl(y|d)=∂αlnλα(d|y)α=0≃∂αlnLα(d|y)α=0=−2Rew+(y|d).

Together with the phase score, this gives an operational decomposition of the complex path weight into quadrature responses. Combining the phase and loss scores gives

(43) $$w_{+}\left(y|d\right)=-\frac{1}{2}s_{l}\left(y|d\right)+\frac{i}{2}s_{\phi }\left(y|d\right).$$

Thus two calibrated perturbations, a phase perturbation and a loss perturbation applied to the same slit, reconstruct the complex upper-path response in source-label space. This is conceptually related to direct optical wavefunction and weak-value reconstruction, but the present object is a source-label-dependent response weight used for fixed-detector TRY sensing rather than a full quantum-state reconstruction [50,51,53].

**Figure 5:**
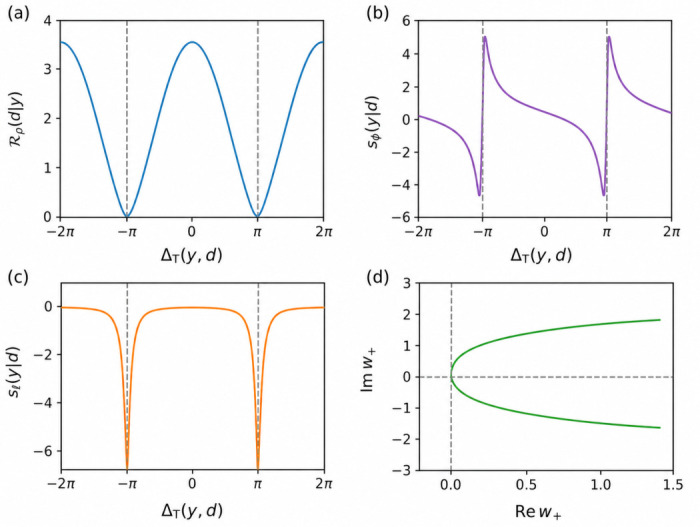
Double-slit TRY response to one-slit perturbations. (**a**) Relative fixed-detector count-rate profile $R_{\rho }\left(d|y\right)=1+\rho^{2}+2\rho \cos\Delta_{T}\left(y,d\right)$ for a two-path model with path-amplitude ratio ρ. (**b**) Phase score $s_{\phi }\left(y|d\right)$, obtained from a weak one-slit phase perturbation and proportional to Imw+. (**c**) Loss score $s_{l}\left(y|d\right)$, obtained from a weak one-slit amplitude-loss perturbation and proportional to −Rew+. (**d**) Complex path-weight trajectory reconstructed from $w_{+}\left(y|d\right)=-\left(1/2\right)s_{l}\left(y|d\right)+\left(i/2\right)s_{\phi }\left(y|d\right)$. The dashed vertical lines in panels (**a**–**c**) mark the balanced-model source-space nulls. For ρ=1, the loss score is constant; for ρ≠1, path imbalance makes the real part of the path response source-label dependent.

Figure 5 shows how this reconstruction works in a slightly imbalanced two-path model. Panel (a) gives the relative fixed-detector count-rate profile $R_{\rho }\left(d|y\right)=1+\rho^{2}+2\rho \cos\Delta_{T}\left(y,d\right)$, where the imbalance parameter ρ prevents the nulls from becoming perfectly dark. Panel (b) shows the phase score $s_{\phi }\left(y|d\right)$, which probes the imaginary part of the upper-path weight and is strongly enhanced near the source-space destructive-interference points. Panel (c) shows the loss score $s_{l}\left(y|d\right)$, which probes the real part of the same path weight and becomes source-label dependent when ρ≠1. Panel (d) combines the two scores into the complex trajectory $w_{+}\left(y|d\right)=-\left(1/2\right)s_{l}\left(y|d\right)+\left(i/2\right)s_{\phi }\left(y|d\right)$. Thus the figure gives a direct diagnostic test: agreement between the measured phase–loss reconstruction and the predicted $w_{+}$ trajectory verifies the two-path response model, while deviations reveal imbalance, finite-aperture effects, partial coherence, or background. This makes the figure a calibration diagnostic as well as a theoretical response plot.

In the perfectly balanced ideal model, Rew+=1/2, so the loss score is spatially constant and carries no normalized-shape information after centering. This does not make the loss scan useless. It contributes to the evidence channel, and in realistic systems with imbalance, finite slit width, partial coherence, or background, Rew+ need not be constant. The loss scan then supplies the real part of the source-space path weight and helps diagnose path imbalance. This role is especially useful in realistic interferometers, where contrast and imbalance are often limited by unequal transmission and partial coherence [30,32].

Figure 5 should be used as the model-to-experiment bridge. It compares the analytic response expected from the calibrated geometry with the measured phase and loss score profiles obtained from the protocol below.

### 5.4. Relation to CYI in the Same Double-Slit System

For the same aperture, CYI with fixed source $y_{0}$ has detector-plane phase

(44) $$\Delta_{Y}\left(x,y_{0}\right)=ka\left(\frac{y_{0}}{z_{1}}+\frac{x}{z_{2}}\right),$$

and detector-plane relative count-rate profile

(45) $$R_{Y}\left(x|y_{0}\right)=4\cos^{2}\;\left[\frac{\Delta_{Y}\left(x,y_{0}\right)}{2}\right].$$

The corresponding CYI click likelihood is again obtained from Equation (7), with $\lambda \left(x|y_{0}\right)=\lambda_{0}R_{Y}\left(x|y_{0}\right)+B$. The local phase score has the same functional form,

(46) $$s_{\phi }^{Y}\left(x|y_{0}\right)=-\tan\;\left[\frac{\Delta_{Y}\left(x,y_{0}\right)}{2}\right].$$

This confirms that the local weak-response formula is not unique to TRY. The same tangent response is the ordinary two-path phase sensitivity near a dark fringe [2,3,41].

The difference is statistical and operational. CYI samples the score landscape by observing detector coordinate *x* for a fixed source $y_{0}$. TRY samples the score landscape by programming source coordinate *y* for a fixed detector *d*. In the ideal symmetric double-slit model these landscapes are related by coordinate exchange, but in practical sensing the available controls are different. TRY allows the source prior to be designed before detection, enabling two-label source coding, posterior balancing, and fixed-detector differential readout. This is the point where TRY differs from both conventional detector-plane sampling and detector-side postselection: the informative ensemble is shaped by source programming before propagation. CYI would require detector scanning, detector-pixel weighting, or detector-side postselection to implement an analogous strategy. The distinction is therefore architectural rather than a claim that the local two-path response formula itself is new.

## 6. Experimental Protocol

The theory above can be tested with a passive double-slit setup and a fixed detector. The essential measurement is simple: record the source-label count histogram before and after a calibrated weak perturbation, then form the score, susceptibility, and Fisher information from count ratios. This section gives a minimal TRY protocol and a CYI control.

**Figure 6:**
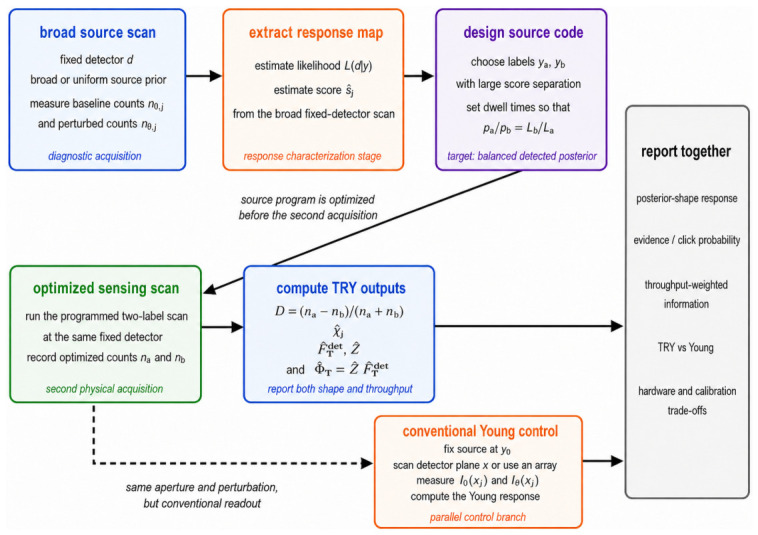
Experimental workflow for source-prior engineering in TRY. A broad fixed-detector source scan with a uniform or broad prior records baseline and perturbed source-label counts, from which the count-rate profile λ(d|y), calibrated click likelihood L(d|y), and score $s_{\theta }\left(y|d\right)$ are estimated. These response maps are then used to design a source code, for example by choosing two labels with large score separation and setting dwell times according to $p_{a}/p_{b}=L_{b}/L_{a}$ to balance the detected posterior. The optimized sensing scan is a second physical acquisition at the same fixed detector and yields the counts used to compute the differential imbalance *D*, centered susceptibility ${\hat{\chi }}_{j}$, detected-event Fisher information ${\hat{F}}_{T}^{\det}$, evidence $\hat{Z}$, and throughput-weighted information ${\hat{\Phi }}_{T}$. A conventional Young control uses the same aperture and perturbation but replaces source-side programming with detector-plane sampling, enabling a direct comparison of posterior-shape response, evidence, throughput, and hardware-calibration trade-offs.

Figure 6 summarizes the experimental workflow. The first stage is a broad fixed-detector source scan, in which a uniform or broad source prior records baseline and perturbed count histograms over source labels. These data are used to estimate the count-rate landscape λ(d|y). For gated on/off operation, the rates are converted through Equation (7) to the click-likelihood landscape L(d|y); for low occupancy, L∝λ. The associated perturbation score is then obtained from the appropriate click or rate model. These quantities are the empirical inputs to the Bayesian design rules derived above. The second stage uses these response maps to choose a source code, for example two labels with large score separation and dwell times satisfying $p_{a}/p_{b}=L_{b}/L_{a}$, so that the detected posterior is balanced. The optimized scan is then a new physical acquisition at the same fixed detector, not a reweighting of the diagnostic data. This distinction is consistent with Bayesian and optimal experimental design, where the next measurement setting is physically chosen using information from an earlier diagnostic measurement [6,39]. It gives the count ratios used to obtain the imbalance *D*, centered susceptibility ${\hat{\chi }}_{j}$, detected-event Fisher information ${\hat{F}}_{T}^{\det}$, evidence $\hat{Z}$, and throughput-weighted information ${\hat{\Phi }}_{T}$. The conventional Young branch uses the same aperture and perturbation but replaces source-side programming with detector-plane sampling, providing the appropriate architecture-level control.

### 6.1. Minimal Fixed-Detector TRY Test

A practical TRY implementation [21] requires a point-addressable source plane, a passive two-path aperture, and one fixed detector. The source may be a single-mode fiber on a translation stage, a pinhole defined by a spatial light modulator, a digital micromirror device, or an addressable emitter array. The detector can be a photodiode, avalanche photodiode, camera pixel, or single-photon detector. These components are standard in phase-measurement interferometry and wavefront-testing implementations [45,46,47].

A calibrated perturbation is applied to one slit. A weak phase can be introduced with a thin glass plate, a piezo-actuated cover slip, a liquid-crystal phase patch, or a calibrated phase mask. A weak loss can be introduced with a neutral-density patch, a partial absorber, or an amplitude-modulating spatial light modulator. The phase perturbation tests the imaginary part of the path response through Equation (37); the loss perturbation tests the real part through Equation (42).

Let the source be addressed at positions $y_{j}$, with programmed dwell times $T_{j}$. In the baseline scan, record fixed-detector counts $n_{0j}$. After applying a weak phase ϵ, record counts $n_{\epsilon j}$ using the same source program. Let $b_{j}$ be the independently measured background count for the same dwell time. The empirical phase score is estimated by

(47) $${\hat{s}}_{\phi ,j}=\frac{1}{\epsilon }\ln\;\left[\frac{n_{\epsilon j}-b_{j}}{n_{0j}-b_{j}}\right].$$

The logarithmic count-ratio form above estimates the count-rate score $\partial_{\phi }\ln\lambda$ and is appropriate when background-subtracted counts are well above noise. It equals the click-likelihood score in the low-occupancy regime. If the per-gate occupancy is not negligible, one should first estimate ${\hat{\lambda }}_{j}=\left(n_{j}-b_{j}\right)/T_{j}$, convert it to ${\hat{L}}_{j}=1-\exp\left(-\tau {\hat{\lambda }}_{j}\right)$, and form the finite-difference logarithm of ${\hat{L}}_{j}$ rather than of the raw count rate. If dwell times are changed between scans, count rates should be used instead of raw counts. The linear-response condition and leading counting uncertainty of this estimator are summarized in Appendix E.

The baseline detected posterior is estimated by

(48) $${\hat{P}}_{0j}=\frac{n_{0j}-b_{j}}{\sum_{k}\left(n_{0k}-b_{k}\right)}.$$

The centered TRY susceptibility and detected-event Fisher information are, respectively,

(49) $${\hat{\chi }}_{\phi ,j}={\hat{s}}_{\phi ,j}-\sum_{k}{\hat{P}}_{0k}{\hat{s}}_{\phi ,k},$$

(50) $${\hat{F}}_{T}^{\det}=\sum_{j}{\hat{P}}_{0j}{\hat{\chi }}_{\phi ,j}^{\;2}.$$

For $N_{d}=\sum_{j}\left(n_{0j}-b_{j}\right)$ detected baseline events, the shot-noise-limited perturbation uncertainty is approximately

(51) $$\delta \epsilon_{\min}≃\frac{1}{\sqrt{N_{d}{\hat{F}}_{T}^{\det}}}.$$

This equation gives a direct experimental scale and explains why conditional Fisher information and detected-event number must be reported together. It is the usual Cramér–Rao scaling for independent count samples [7,8,23].

### 6.2. Complex Path-Response Reconstruction

To reconstruct the complex response, repeat the measurement with a weak amplitude loss α on the same slit. Similarly, the empirical loss score is

(52) $${\hat{s}}_{l,j}=\frac{1}{\alpha }\ln\;\left[\frac{n_{\alpha j}-b_{j}}{n_{0j}-b_{j}}\right],$$

where $n_{\alpha j}$ is the count after the loss perturbation. The complex upper-path response is then reconstructed as

(53) $${\hat{w}}_{+,j}=-\frac{1}{2}{\hat{s}}_{l,j}+\frac{i}{2}{\hat{s}}_{\phi ,j},$$

following Equation (43). This reconstruction checks the internal consistency of the response model and reveals imperfections such as path imbalance, finite-aperture effects, partial coherence, or background-induced distortions. It is therefore analogous in spirit to weak-value or direct-measurement consistency checks, while remaining a calibrated TRY response measurement rather than a state-reconstruction protocol [50,51,53].

### 6.3. Adaptive Source-Prior Engineering

The fixed-detector measurement naturally supports a two-stage protocol. First, perform a coarse scan with a broad source prior to estimate the background-corrected rates ${\hat{\lambda }}_{j}=\left(n_{0j}-b_{j}\right)/T_{j}$. For gated on/off data, convert them to ${\hat{L}}_{j}=1-\exp\left(-\tau {\hat{\lambda }}_{j}\right)$; in the low-occupancy regime, ${\hat{L}}_{j}≃\tau {\hat{\lambda }}_{j}$. The perturbation scores ${\hat{s}}_{\phi ,j}$ are estimated from the same scan. Second, choose labels with large score separation and acceptable likelihood. For two labels a,b, choose dwell times

(54) $$\frac{T_{a}}{T_{b}}=\frac{{\hat{L}}_{b}}{{\hat{L}}_{a}},$$

which implements the prior-ratio rule in Equation (24) for low-occupancy gated clicks. More generally, the right-hand side is ${\hat{L}}_{b}/{\hat{L}}_{a}$ for Bernoulli click trials and ${\hat{\lambda }}_{b}/{\hat{\lambda }}_{a}$ when balancing unthresholded Poisson counts. The predicted detected-event Fisher information is

(55) $${\hat{F}}_{2}^{\det}=\frac{{\left({\hat{s}}_{\phi ,a}-{\hat{s}}_{\phi ,b}\right)}^{2}}{4}.$$

The corresponding differential readout is the label imbalance *D* in Equation (26).

This second-stage measurement is not post-processing of the initial data. It physically reallocates source exposure before detection so that the fixed detector samples the most informative source labels. In this sense, the TRY protocol realizes an optical version of sequential experimental design: a diagnostic acquisition determines the physical settings of the optimized acquisition [38,40]. This is the operational point that connects TRY to experimental design: the data from the first stage determine the physical settings of the second stage [6,39], i.e., the experimental meaning of source-prior engineering.

### 6.4. Throughput and Noise Checks

A practical demonstration using gated on/off trials should report ${\hat{F}}_{T}^{\det}$, the estimated click probability $\hat{Z}=N_{\mathrm{click}}/N_{\mathrm{trial}}$, and the throughput-weighted value ${\hat{\Phi }}_{T}=\hat{Z}{\hat{F}}_{T}^{\det}$. For unthresholded Poisson-count acquisition, the corresponding quantities are the mean detected rate $\hat{\bar{\lambda }}$ and the posterior-shape information rate $\hat{\bar{\lambda }}{\hat{F}}_{T,\lambda }^{\det}$. Reporting ${\hat{F}}_{T}^{\det}$ alone can be misleading near nulls, because the detected-event information may increase while the number of detected events decreases. The same conditional-information versus event-probability tradeoff appears in postselected and weak-value-enhanced measurements [33,34,37].

The perturbation should also be small enough that the score estimate is linear. A useful check is to repeat the measurement for two perturbation magnitudes, for example ϵ and ϵ/2, and verify that the inferred score is unchanged within uncertainty. Background subtraction should be performed before taking logarithms. Source positions with counts comparable to background should be excluded or treated with a full Poisson likelihood model rather than the simple log-ratio estimator. This is the standard remedy when Gaussian or logarithmic approximations to count statistics fail [8,23].

### 6.5. Calibration, Stability, and Robustness to Experimental Imperfections

A useful experimental count-rate model that includes the dominant nonidealities is

(56) $${\tilde{\lambda }}_{j}=G_{j}\eta_{j}\;\left[I_{+,j}+I_{-,j}+2\nu_{j}\sqrt{I_{+,j}I_{-,j}}\cos\left(\Delta_{j}+\phi \right)\right]+B_{j},$$

where $G_{j}\eta_{j}$ is the combined source–detector gain, $B_{j}$ is additive background, and

(57) $$\nu_{j}=\left|\gamma_{+-,j}\right|\;|e_{+,j}^{†}e_{-,j}|\leq 1$$

combines first-order spatial/temporal coherence and polarization overlap [30,32]. Equation (56) makes the practical requirements explicit. The corresponding per-trial click likelihood is ${\tilde{L}}_{j}=1-\exp\left(-\tau {\tilde{\lambda }}_{j}\right)$. A gain factor common to all labels cancels from the normalized posterior and from the ratio *D*, but label-dependent gain, source-position-dependent coupling, detector nonlinearity, or temporal drift can imitate a posterior-shape response. The source labels should therefore be interleaved or randomized on a timescale shorter than the dominant drift, and the detector dark count, linearity, and gain should be calibrated independently. A reference source label away from the null can be revisited periodically to monitor residual gain drift.

Let the implemented source prior contain small relative errors,

(58) $${\tilde{p}}_{j}=\frac{p_{j}\left(1+\varepsilon_{j}\right)}{\sum_{k}p_{k}\left(1+\varepsilon_{k}\right)}.$$

To first order, the induced posterior error is itself centered,

(59) $$\delta \ln P_{j}=\varepsilon_{j}-{\langle \varepsilon \rangle }_{P}+O\left(\varepsilon^{2}\right).$$

For a nominally balanced two-label code, this gives a zero-signal imbalance and an equivalent parameter bias

(60) $$D_{0}≃\frac{\varepsilon_{a}-\varepsilon_{b}}{2},\;\delta \theta_{\mathrm{prog}}≃\frac{\varepsilon_{a}-\varepsilon_{b}}{s_{a}-s_{b}}.$$

Thus, the relevant source-programming specification is the differential dwell-time or brightness error, not the absolute source power. The same expression applies to differential detector gain drift. Source-position uncertainty $\sigma_{y}$ should additionally satisfy $|\partial_{y}s_{j}|\sigma_{y}$ below the desired score uncertainty at each selected label. Uncertainty in the calibrated perturbation amplitude rescales the score estimate and should be propagated as a multiplicative calibration uncertainty.

**Figure 7:**
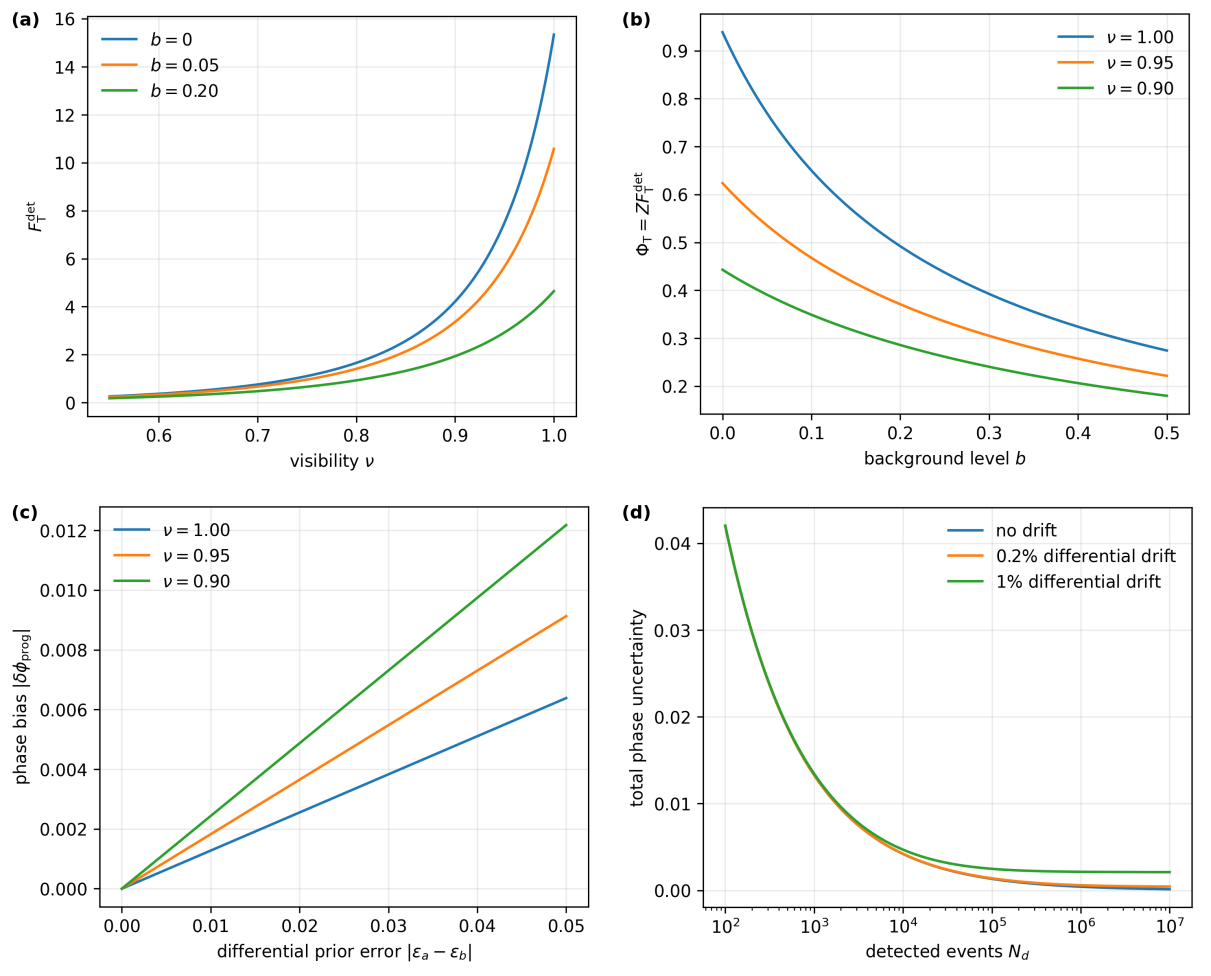
Illustrative robustness analysis for a symmetric two-label TRY phase sensor. (**a**) Detected-event Fisher information versus visibility ν for several background levels *b*. (**b**) Normalized throughput-weighted information $\Phi_{T}/\kappa$ versus background for several visibilities. (**c**) Equivalent phase bias versus differential source-prior error, as predicted by Equation (60). (**d**) Total phase uncertainty versus detected-event number, showing the transition from shot-noise scaling to floors produced by differential drift. The labels are located at Δ=±(π−0.5); all quantities are dimensionless and are intended to show trends rather than a hardware-specific sensitivity.

Figure 7 gives an illustrative robustness study for a symmetric two-label code with labels Δ=±(π−δ), δ=0.5 rad, and the normalized response profile r=[2+2νcosΔ+b]/(4+b). The weak-occupancy click likelihood is L=κr, where the calibrated peak click probability κ≤1 sets the absolute throughput; panel (b) therefore reports $\Phi_{T}/\kappa$. The visibility ν represents either partial coherence or polarization mismatch, and *b* is a background level relative to one-path intensity. Reduced visibility and increased background regularize the near-null score and lower both conditional and throughput-weighted information. Differential prior error produces the linear bias predicted by Equation (60). Finally, shot-noise improvement with increasing event number saturates once differential drift establishes a systematic floor. The calculation is not a hardware-specific forecast; it identifies the calibration quantities that must be reported in a proof-of-principle experiment.

### 6.6. Conventional Young Control

A fair control uses the same double slit and calibrated perturbation, but fixes the source at $y_{0}$ and measures the detector-plane response. This control is the standard detector-plane Young measurement against which the source-side TRY architecture should be compared [1,2,3]. This control branch is also consistent with established phase-demodulation and quadrature-readout strategies in precision laser interferometry [48,54]. If a detector array is available, the baseline and perturbed intensities $I_{0}\left(x_{j}\right)$ and $I_{\epsilon }\left(x_{j}\right)$ give

(61) $${\hat{s}}_{\phi ,j}^{Y}=\frac{1}{\epsilon }\ln\;\left[\frac{I_{\epsilon }\left(x_{j}\right)-b_{j}}{I_{0}\left(x_{j}\right)-b_{j}}\right],$$

and

(62) $${\hat{F}}_{Y}^{\det}=\sum_{j}{\hat{P}}_{0j}^{Y}{\left({\hat{s}}_{\phi ,j}^{Y}-\sum_{k}{\hat{P}}_{0k}^{Y}{\hat{s}}_{\phi ,k}^{Y}\right)}^{2},$$

where ${\hat{P}}_{0j}^{Y}$ is the normalized baseline detector histogram.

The comparison should not be framed as a universal sensitivity ranking. In the ideal symmetric double-slit model, the local phase-score profiles of traditional Young and TRY have the same functional form under coordinate exchange. The relevant comparison is operational: CYI uses detector-plane sampling or detector-array readout, whereas TRY uses source-side programming and a fixed detector. A meaningful experiment should compare not only Fisher information, but also throughput, calibration stability, background sensitivity, and the availability of source-side versus detector-side programmability. Such an architecture-level comparison is essential because local amplification alone does not determine experimental sensitivity [33,34]. This architecture-level comparison is also familiar from coded and single-pixel optical systems, where the measurement code, detector architecture, and reconstruction or estimation task must be assessed together [18,19,49,55].

## 7. Discussion

The Bayesian formulation developed above gives TRY a role different from conventional detector-plane Young sensing and postselected weak-response measurements [10,12,33,34]. The posterior, score, and Fisher-information identities are standard [7,8,9,23]. The new physical feature is that TRY makes the source prior an optical control applied before propagation, extending the fixed-detector source-space viewpoint established previously [21]. This converts prior design into a way of shaping the detected response ensemble at a fixed detector.

This distinction is important for interpreting sensitivity. A large local score near a source-space null is not by itself a metrological advantage, as is well known from Fisher-information analyses of amplified and postselected measurements [33,34,37]. The same null can strongly reduce the evidence, so the number of detected events may fall. The useful quantity is therefore not the peak value of the score, but the posterior-weighted score variance together with the evidence. This is why $F_{T}^{\det}$, *Z*, and $ZF_{T}^{\det}$ should be reported together. They separate conditional response enhancement from the cost of obtaining the conditioned events.

The two-label protocol gives the most transparent physical realization of this principle. It does not require reconstruction of a full field or a full source-space fringe. A preliminary scan identifies two labels with large score separation and acceptable likelihood. A second measurement balances their detected posterior weights and reads out the perturbation through a label imbalance. This two-stage structure follows the logic of experimental design, but here the optimized setting is the source prior itself [6,39]. In this form, TRY becomes a fixed-detector differential sensor. Its advantage is not universal higher Fisher information, but a different hardware allocation: source-side coding replaces detector-plane scanning or detector-array weighting.

The multiparameter covariance form suggests a broader application. In realistic interferometers, the desired perturbation often appears together with nuisance effects such as loss, source-power drift, aperture imbalance, defocus, or pointing error. Since the Fisher matrix is the covariance of score functions over the detected posterior, the source program can be chosen to make the target score distinguishable from nuisance scores, following the standard Fisher-matrix treatment of multiparameter estimation with nuisance parameters [8,9,23]. This turns TRY from a single-parameter response measurement into a compact source-coded design problem. The rank bound further shows how many source labels are needed to distinguish a given number of independent perturbation directions.

Several limitations should be emphasized. First, the closed-form double-slit expressions assume a scalar, coherent, paraxial model with narrow slits. Finite aperture width, partial coherence, polarization mismatch, background, and detector saturation modify the likelihood and score landscape [2,30,32]. These effects do not invalidate the Bayesian structure, but they must be included through a calibrated count-rate-to-click model or a direct Poisson count model. Second, near-null operation is sensitive to background and drift, a common limitation in dark-port or postselected response enhancement [33,34,52]; labels with counts comparable to background should not be used with simple logarithmic estimators. Third, source-programming error and label-dependent gain drift create a direct bias according to Equation (60). The practical comparison with CYI therefore depends on the relative quality of source-side programmability and detector-side spatial readout. Section 6 and Figure 7 now provide explicit calibration and robustness criteria rather than treating these effects only qualitatively.

The most useful interpretation is one of complementarity. CYI measures a detector-coordinate response from a fixed source, while TRY measures a source-label posterior response at a fixed detector. These are different statistical projections of the same passive optical kernel. Which one is preferable depends on the perturbation, the available hardware, and the dominant technical noise. TRY is especially attractive when detector scanning, detector arrays, or detector flat-field calibration are costly, while source programming is accurate and flexible.

### Generalization to Multi-Aperture and Integrated Photonic Systems

The framework is not restricted to a two-slit aperture. For an *M*-path interferometer,

(63) A(d,y;θ)=∑m=1MAm(d,y;θ),sθμ(y|d)=2Re∂θμAA

for an ideal intensity likelihood. Multi-slit interferometers and diffraction gratings therefore require no change in the Bayesian construction; they merely generate higher-dimensional and often periodic score landscapes. The source prior can select labels near different grating orders, Talbot-like revivals, or mask-dependent response features, while the rank condition in Equation (31) determines how many independent perturbations can be separated [3,26,29].

A structured phase or amplitude mask remains covered by the continuous propagation integral in Equation (4). The same idea also maps directly to an integrated photonic circuit with programmable input ports *j*, a fixed monitored output port *r*, and transfer matrix U(θ):

(64) $$P\left(j|r;\theta \right)=\frac{p_{j}{\left|\langle r|U(\theta )|j\rangle \right|}^{2}}{\sum_{k}p_{k}{\left|\langle r|U(\theta )|k\rangle \right|}^{2}}.$$

Here the input-port probabilities or launch times implement the prior, and one photodiode or single-photon detector monitors the fixed output port. Tunable meshes, multimode interferometers, ring-resonator networks, and reconfigurable phase shifters can therefore realize the same source-prior design principle on chip [56]. Structured illumination and quantitative phase imaging are additional natural application contexts because controlled illumination or phase-sensitive readout already encodes otherwise inaccessible information into intensity data [57,58]. In every case, the operational task is unchanged: calibrate the likelihood or score landscape, select an informative source support, and preserve sufficient evidence.

## 8. Conclusions

In summary, time-reversed Young interferometry has been formulated as a source-coded Bayesian response sensor. In this view, the fixed-detector source-label histogram is a posterior distribution, and a weak optical perturbation moves this posterior through its likelihood score. The resulting TRY susceptibility is the centered posterior score, while the detected-event Fisher information is the posterior score variance. These identities are standard in estimation theory, but TRY gives them a distinct physical role because the source prior is programmed before propagation and therefore controls the response ensemble actually sampled by the fixed detector.

The framework leads to several practical results. It separates posterior-shape information from evidence, making clear why near-null response enhancement must be evaluated together with click probability. It gives a two-label differential source code in which a perturbation is read out as a fixed-detector source-label imbalance. It extends naturally to multiparameter sensing, where the posterior covariance of score functions provides a design rule for nuisance rejection. It also gives a minimal-label rank condition: posterior-shape sensing of *m* independent perturbation directions requires at least m+1 occupied source labels.

A passive double-slit system with a weak one-slit phase or loss perturbation provides a direct experimental test. The necessary data are fixed-detector source scans before and after a calibrated perturbation, from which the score, susceptibility, Fisher information, and complex path response can be reconstructed. The added robustness analysis specifies how source-prior error, gain drift, background, imperfect coherence, and polarization mismatch enter the measurement, while the multiport formulation extends the method to multi-aperture and integrated photonic systems. The comparison with CYI is not a claim of universal sensitivity enhancement. Rather, TRY realizes a complementary statistical experiment: source-side posterior response engineering with a fixed detector. This makes TRY a practical platform for source-coded optical sensing when source programming is more accessible or stable than detector-plane scanning or detector-array calibration.

## Figures and Tables

**Table 1:** Typical target and nuisance perturbations in a TRY measurement. Here $A=A_{+}+A_{-}$, $w_{+}=A_{+}/A$, $I_{\pm }={\left|A_{\pm }\right|}^{2}$, $L_{0}=I_{+}+I_{-}$, $C=2\sqrt{I_{+}I_{-}}\cos\Delta$, and *L* denotes the total likelihood.

Perturbation	Likelihood Modification	Local Score	Consequence and Treatment
One-path phase ϕ	$A_{+}\;\to e^{-i\phi }A_{+}$	sϕ=2Imw+	Desired phase signal or phase-drift nuisance; use labels with strong phase-score contrast.
One-path loss *ℓ*	$A_{+}\;\to e^{-l}A_{+}$	sl=−2Rew+	Estimates path loss and diagnoses imbalance; include jointly with phase when necessary.
Common power or gain *g*	$L\;\to e^{g}L$	$s_{g}=1$	Centered score vanishes; affects evidence but not normalized posterior shape.
Path imbalance ρ	$A=A_{+}+\rho A_{-}$	sρ=2Re(A−/A)	Changes both fringe visibility and score geometry; calibrate or treat as a nuisance parameter.
Coherence/polarization visibility ν	$L=L_{0}+\nu C+B$	$s_{\nu }=C/L$	Partial coherence and polarization mismatch reduce contrast and near-null response.
Additive background *B*	$L=L_{\mathrm{sig}}+B$	$s_{B}=1/L$	Dominant near dark fringes; subtract independently or use a full Poisson likelihood.
Defocus or pointing *q*	A→A(q)	sq=2Re[(∂qA)/A]	Produces a label-dependent nuisance direction; use at least three labels for joint phase–nuisance sensing.

**Table 2:** Qualitative comparison of representative interferometric and coded optical-sensing architectures.

Architecture	Programmed or Scanned Degree of Freedom	Principal Strength	Main Trade-Off Relative to TRY
CYI with camera or detector scan	Detector coordinate	Direct spatial fringe acquisition and high parallel throughput with a camera	Requires detector-plane resolution, scanning, or pixel calibration.
Phase-stepped or heterodyne interferometry	Reference phase or local oscillator	Accurate phase and quadrature recovery	Requires controlled phase steps, phase locking, or demodulation calibration.
Dark-port/weak-value sensing	Detector-side postselection	Large conditional response and possible benefit under saturation or restricted detector range	Success-probability penalty and sensitivity to background and drift near the null.
Single-pixel or computational imaging	Illumination or mask patterns	Replaces detector arrays with programmable coding and one detector	Usually reconstructs an object/image from many patterns and a computational inverse problem.
TRY source-prior sensing	Source-label prior before propagation	Fixed-detector posterior engineering, differential label readout, and source-side nuisance control	Requires accurate source positioning, dwell-time calibration, and sufficient evidence at selected labels.

## Data Availability

No experimental datasets were generated in this study. The numerical robustness curves are fully determined by the analytical model and parameter values stated in the manuscript.

## Abbreviations

The following abbreviations are used in this manuscript:

TRY	time-reversed Young
CYI	conventional Young interferometry
FI	Fisher information
SLM	spatial light modulator
DMD	digital micromirror device

## Appendix A. Centered Posterior Score

Starting from Equation (1), with a perturbation-independent prior, the logarithmic derivative is

(A1) $$\partial_{\theta }\ln P=\partial_{\theta }\ln L-\partial_{\theta }\ln Z.$$

The first term is the likelihood score. For the second term,

(A2) $$\begin{array}{cc} \partial_{\theta }Z & =\int dy\;p\left(y\right)\partial_{\theta }L\left(d|y;\theta \right)\\ \; & =\int dy\;p\left(y\right)L\left(d|y;\theta \right)s_{\theta }\left(y|d\right)\\ \; & =Z{\langle s_{\theta }\rangle }_{P}. \end{array}$$

Therefore $\partial_{\theta }\ln Z={\langle s_{\theta }\rangle }_{P}$, proving Equation (16). Substituting the same centered score into the Fisher-information definition gives Equation (17).

## Appendix B. Evidence and Trial-Level Fisher Information

For a discrete source program, let $\pi_{j}=p_{j}L_{j}$ be the probability that one source trial produces a fixed-detector click with label *j*. The no-click probability is 1−Z, with $Z=\sum_{j}\pi_{j}$. The per-trial Fisher information for the joint click/no-click outcome is

(A3) $$F_{\mathrm{trial}}=\sum_{j}\frac{{\left(\partial_{\theta }\pi_{j}\right)}^{2}}{\pi_{j}}+\frac{{\left(\partial_{\theta }Z\right)}^{2}}{1-Z}.$$

This is the standard classical Fisher information for a discrete probability distribution [7,8,23]. Using $\partial_{\theta }\pi_{j}=\pi_{j}s_{j}$ and $\partial_{\theta }Z=Z{\langle s\rangle }_{P}$, Equation (A3) becomes

(A4) $$F_{\mathrm{trial}}=Z{\langle s^{2}\rangle }_{P}+\frac{Z^{2}{\langle s\rangle }_{P}^{2}}{1-Z}=Z{\mathrm{Var}}_{P}\left(s\right)+\frac{Z{\langle s\rangle }_{P}^{2}}{1-Z},$$

which is Equation (19).

For completeness, consider independent Poisson counts $N_{j}$ collected during a total exposure *T*, with mean $\Lambda_{j}=Tp_{j}\lambda_{j}$. Their Fisher information is

(A5) $$\begin{array}{cc} F_{\mathrm{Pois}}\left(T\right)\; & =\sum_{j}\frac{{\left(\partial_{\theta }\Lambda_{j}\right)}^{2}}{\Lambda_{j}}=T\sum_{j}p_{j}\frac{{\left(\partial_{\theta }\lambda_{j}\right)}^{2}}{\lambda_{j}} \end{array}$$

(A6) $$\begin{array}{cc} \; & =T\bar{\lambda }\left[{\mathrm{Var}}_{P_{\lambda }}\left(r_{\theta }\right)+{\langle r_{\theta }\rangle }_{P_{\lambda }}^{2}\right], \end{array}$$

where $P_{\lambda ,j}=p_{j}\lambda_{j}/\bar{\lambda }$, $\bar{\lambda }=\sum_{j}p_{j}\lambda_{j}$, and $r_{\theta }=\partial_{\theta }\ln\lambda_{j}$. The first term is the information in the normalized distribution of detected count labels, and the second is the information in the total Poisson rate. With $L_{j}=1-\exp\left(-\tau \lambda_{j}\right)$, the Bernoulli result approaches $F_{\mathrm{trial}}=\tau F_{\mathrm{Pois}}^{\left(\mathrm{rate}\right)}+O\left(\mu_{\max}^{2}\right)$ in the low-occupancy limit, where $\mu_{\max}=\max_{j}\left(\tau \lambda_{j}\right)$. Thus the posterior-shape theory is common to both detection models, provided that *L* is used for gated clicks and λ is used for unthresholded counts.

## Appendix C. Discrete Source-Prior Design and Rank Bound

For selected labels with nonzero likelihood, any desired posterior $q_{j}$ can be realized by choosing

(A7) $$p_{j}=\frac{q_{j}/L_{j}}{\sum_{k}q_{k}/L_{k}}.$$

This relation gives the dwell-time or brightness program needed to implement a target detected posterior.

For two labels, equal detected posterior weights imply Equation (24). If only those two labels are used, the evidence is Equation (25). The detected-event Fisher information is Equation (22); maximizing it over $P_{a}$ gives Equation (23). For more labels with bounded scores, the largest possible variance is obtained by placing posterior weight on the two extreme scores.

For multiple perturbations, each occupied label defines a score vector $s_{j}$. The Fisher matrix is the covariance of these vectors. Centering removes one weighted mean vector, so *J* occupied labels span at most J−1 independent centered directions. This proves Equation (31).

## Appendix D. Double-Slit Phase and Loss Scores

Let $A=A_{+}+A_{-}$ and $w_{+}=A_{+}/A$. The ratio $w_{+}$ has the same normalized conditional-amplitude structure as a path weak value, although here it is used as a calibrated TRY response weight rather than as a state-reconstruction object [10,11,12]. For the phase perturbation in Equation (35), $\partial_{\epsilon }A_{\epsilon }=-iA_{+}$ at ϵ=0. Therefore,

(A8) ∂ϵln|Aϵ|2=2Re−iA+A=2Imw+.

For the loss perturbation in Equation (41), $\partial_{\alpha }A_{\alpha }=-A_{+}$, giving

(A9) ∂αln|Aα|2=−2Rew+.

Combining the two scores gives Equation (43).

For fitting real data, it is useful to allow path imbalance. Let $A_{-}/A_{+}=\rho e^{i\Delta }$, where ρ is the path-amplitude ratio. Then

(A10) $$w_{+}=\frac{1+\rho \cos\Delta }{D}-i\frac{\rho \sin\Delta }{D},\;D=1+\rho^{2}+2\rho \cos\Delta .$$

The corresponding count-rate scores, equal to the click-likelihood scores in the low-occupancy limit, are

(A11) $$s_{\phi }=-\frac{2\rho \sin\Delta }{D},\;s_{l}=-\frac{2(1+\rho \cos\Delta )}{D}.$$

The balanced case in the main text follows from ρ=1. Equation (A11) is useful when unequal slit transmission, finite-aperture effects, or partial coherence make the response deviate from the ideal model.

## Appendix E. Count-Ratio Score Estimators

The experimental score estimators in Section 6 are finite-difference approximations to logarithmic count-rate derivatives. This follows the standard likelihood-score treatment of count-based estimation [8,23]. Let $R_{j}\left(\theta \right)=\lambda_{j}\left(\theta \right)$ be the background-subtracted count rate for source label *j*, and define $r_{j}=\partial_{\theta }\ln\lambda_{j}$. For a small perturbation δθ,

(A12) $$R_{j}\left(\theta +\delta \theta \right)=R_{j}\left(\theta \right)\left[1+\delta \theta \;r_{j}+O\left(\delta \theta^{2}\right)\right].$$

Hence

(A13) $$\frac{1}{\delta \theta }\ln\frac{R_{j}\left(\theta +\delta \theta \right)}{R_{j}\left(\theta \right)}=r_{j}+O\left(\delta \theta \right).$$

In the low-occupancy limit, Equation (9) gives $r_{j}=s_{j}+O\left(\mu_{j}\right)$, which justifies the log-count-ratio estimator for the click-likelihood score. For non-negligible occupancy, the exact finite-difference click score is obtained by converting each rate to $L_{j}=1-\exp\left(-\tau R_{j}\right)$ before taking the logarithmic ratio.

For independent Poisson counts with sufficiently large background-subtracted signals, the leading error-propagation variance is approximately [8,30,32]

(A14) $$\mathrm{Var}\left({\hat{r}}_{j}\right)≃\frac{1}{\delta \theta^{2}}\left(\frac{1}{n_{\delta \theta ,j}-b_{j}}+\frac{1}{n_{0j}-b_{j}}\right).$$

Labels very close to a null are therefore fragile: their scores may be large, but their rate-score estimates can be noisy and background-sensitive. Such labels should be excluded or treated with a full Poisson likelihood model.
